# Supplementary material for: Hot spot prediction in protein-protein interactions by an ensemble system
Source: BMC Syst Biol. 2018 Dec 31;12(Suppl 9):132. doi: 10.1186/s12918-018-0665-8 (PMC6311905; doi:10.1186/s12918-018-0665-8)
Supplement: Supplementary file 2 — All relevant MATLAB code. A simple Matlab implement of our predictor. (ZIP 19,742 kb) [file 12918_2018_665_MOESM2_ESM.zip › Additional file 2/Read ASA/ASA website/dbMPIKT/3Q3JB.html]

 NetSurfP - Protein Surface Accessibility and Secondary Structure Predictions

  

|  |  |  |
| --- | --- | --- |
|  |  | NetSurfP - Protein Surface Accessibility and Secondary Structure Predictions  Technical University of Denmark |

  


---

```
# For publication of results, please cite:
# A generic method for assignment of reliability scores applied to solvent accessibility predictions.
# Bent Petersen, Thomas Nordahl Petersen, Pernille Andersen, Morten Nielsen and Claus Lundegaard
# BMC Structural Biology 2009, 9:51 doi:10.1186/1472-6807-9-51
#
# Column 1: Class assignment - B for buried or E for Exposed - Threshold: 25% exposure, but not based on RSA
# Column 2: Amino acid
# Column 3: Sequence name
# Column 4: Amino acid number
# Column 5: Relative Surface Accessibility - RSA
# Column 6: Absolute Surface Accessibility
# Column 7: Z-fit score for RSA prediction
# Column 8: Probability for Alpha-Helix
# Column 9: Probability for Beta-strand
# Column 10: Probability for Coil
E M  3Q3JB                  1    0.772 154.397  -1.203   0.003   0.003   0.994
E H  3Q3JB                  2    0.577 104.884  -1.470   0.018   0.047   0.935
E H  3Q3JB                  3    0.608 110.559  -0.807   0.053   0.043   0.903
E H  3Q3JB                  4    0.531  96.534  -1.885   0.052   0.084   0.864
E H  3Q3JB                  5    0.371  67.449  -0.391   0.113   0.087   0.800
B H  3Q3JB                  6    0.276  50.168  -1.477   0.052   0.084   0.864
B H  3Q3JB                  7    0.266  48.385  -1.234   0.113   0.087   0.800
E S  3Q3JB                  8    0.445  52.177  -2.121   0.113   0.043   0.844
E S  3Q3JB                  9    0.367  43.012  -1.330   0.053   0.043   0.903
E G  3Q3JB                 10    0.591  46.480  -1.198   0.058   0.017   0.925
E R  3Q3JB                 11    0.491 112.485  -0.196   0.058   0.017   0.925
E E  3Q3JB                 12    0.630 110.009  -0.530   0.053   0.043   0.903
E N  3Q3JB                 13    0.529  77.402  -0.557   0.056   0.142   0.802
E L  3Q3JB                 14    0.304  55.607  -0.931   0.125   0.227   0.648
E Y  3Q3JB                 15    0.437  93.430  -0.757   0.135   0.317   0.548
E F  3Q3JB                 16    0.416  83.411  -1.086   0.064   0.216   0.721
E Q  3Q3JB                 17    0.564 100.730  -1.114   0.052   0.084   0.864
E G  3Q3JB                 18    0.590  46.425  -2.366   0.018   0.047   0.935
E R  3Q3JB                 19    0.575 131.606  -0.705   0.018   0.019   0.964
E A  3Q3JB                 20    0.459  50.637  -1.287   0.018   0.019   0.964
E P  3Q3JB                 21    0.680  96.535  -0.379   0.058   0.017   0.925
E Q  3Q3JB                 22    0.675 120.609  -0.004   0.115   0.016   0.868
E P  3Q3JB                 23    0.585  82.941  -0.007   0.115   0.016   0.868
E V  3Q3JB                 24    0.269  41.361   0.102   0.113   0.043   0.844
E V  3Q3JB                 25    0.337  51.766   0.165   0.020   0.205   0.775
B A  3Q3JB                 26    0.184  20.277   1.257   0.004   0.514   0.481
E R  3Q3JB                 27    0.276  63.135   1.108   0.002   0.816   0.182
B C  3Q3JB                 28    0.045   6.346   1.550   0.001   0.900   0.099
B K  3Q3JB                 29    0.074  15.140   0.942   0.001   0.900   0.099
B L  3Q3JB                 30    0.016   2.875   1.770   0.001   0.959   0.040
B V  3Q3JB                 31    0.020   3.135   1.409   0.000   0.983   0.017
B L  3Q3JB                 32    0.016   2.930   1.553   0.000   0.983   0.017
B V  3Q3JB                 33    0.025   3.781   1.008   0.001   0.959   0.040
B G  3Q3JB                 34    0.045   3.534  -0.486   0.004   0.420   0.576
B D  3Q3JB                 35    0.202  29.094  -0.284   0.005   0.045   0.951
E V  3Q3JB                 36    0.273  42.022  -1.081   0.018   0.019   0.964
B Q  3Q3JB                 37    0.235  41.935  -1.259   0.058   0.017   0.925
B C  3Q3JB                 38    0.047   6.585   0.323   0.257   0.016   0.727
B G  3Q3JB                 39    0.039   3.077  -0.854   0.339   0.016   0.645
B K  3Q3JB                 40    0.104  21.434   0.258   0.751   0.050   0.199
B T  3Q3JB                 41    0.073  10.097  -0.435   0.831   0.044   0.125
B A  3Q3JB                 42    0.045   4.992   0.174   0.911   0.033   0.057
B M  3Q3JB                 43    0.035   7.044   0.050   0.950   0.028   0.022
B L  3Q3JB                 44    0.025   4.504   0.447   0.950   0.028   0.022
B Q  3Q3JB                 45    0.144  25.647   0.902   0.950   0.028   0.022
B V  3Q3JB                 46    0.105  16.200   1.082   0.950   0.028   0.022
B L  3Q3JB                 47    0.077  14.044   0.210   0.911   0.033   0.057
B A  3Q3JB                 48    0.069   7.582   0.594   0.879   0.010   0.111
E K  3Q3JB                 49    0.535 110.008   1.223   0.622   0.015   0.363
E D  3Q3JB                 50    0.607  87.512  -0.013   0.176   0.004   0.820
E C  3Q3JB                 51    0.512  71.913   1.359   0.058   0.017   0.925
B Y  3Q3JB                 52    0.065  13.933  -0.017   0.018   0.019   0.964
E P  3Q3JB                 53    0.450  63.869   1.533   0.018   0.019   0.964
E E  3Q3JB                 54    0.696 121.539  -0.372   0.018   0.047   0.935
E T  3Q3JB                 55    0.662  91.792   0.209   0.052   0.084   0.864
B Y  3Q3JB                 56    0.232  49.664   0.639   0.056   0.142   0.802
E V  3Q3JB                 57    0.486  74.667   0.722   0.056   0.142   0.802
B P  3Q3JB                 58    0.161  22.789   0.265   0.118   0.150   0.732
B T  3Q3JB                 59    0.153  21.221   0.173   0.135   0.317   0.548
B V  3Q3JB                 60    0.110  16.953   0.833   0.159   0.516   0.325
B F  3Q3JB                 61    0.143  28.780  -0.656   0.159   0.516   0.325
B E  3Q3JB                 62    0.161  28.074  -0.039   0.074   0.484   0.442
B N  3Q3JB                 63    0.313  45.896  -0.548   0.023   0.655   0.322
B Y  3Q3JB                 64    0.106  22.738   0.884   0.018   0.846   0.136
E T  3Q3JB                 65    0.351  48.753   1.098   0.001   0.959   0.040
E A  3Q3JB                 66    0.259  28.520   1.659   0.001   0.959   0.040
E C  3Q3JB                 67    0.423  59.361   1.548   0.000   0.983   0.017
B L  3Q3JB                 68    0.071  12.945   1.178   0.000   0.983   0.017
E E  3Q3JB                 69    0.495  86.459   0.697   0.001   0.959   0.040
B T  3Q3JB                 70    0.130  18.059   0.912   0.001   0.900   0.099
E E  3Q3JB                 71    0.717 125.207   0.567   0.004   0.085   0.910
E E  3Q3JB                 72    0.815 142.380   0.268   0.003   0.003   0.994
E Q  3Q3JB                 73    0.536  95.801   1.347   0.003   0.718   0.279
E R  3Q3JB                 74    0.431  98.768   2.148   0.001   0.959   0.040
B V  3Q3JB                 75    0.046   7.086   1.280   0.006   0.962   0.032
E E  3Q3JB                 76    0.317  55.397   2.520   0.006   0.962   0.032
B L  3Q3JB                 77    0.029   5.237   0.785   0.006   0.962   0.032
B S  3Q3JB                 78    0.114  13.314   0.981   0.011   0.918   0.071
B L  3Q3JB                 79    0.028   5.163   0.687   0.011   0.918   0.071
B W  3Q3JB                 80    0.084  20.202   0.791   0.021   0.756   0.223
B D  3Q3JB                 81    0.076  11.009   0.131   0.021   0.451   0.528
B T  3Q3JB                 82    0.123  17.116  -0.035   0.118   0.150   0.732
B S  3Q3JB                 83    0.154  18.049  -0.483   0.113   0.043   0.844
E G  3Q3JB                 84    0.290  22.784  -0.826   0.181   0.016   0.803
B S  3Q3JB                 85    0.234  27.448   0.289   0.522   0.016   0.462
E P  3Q3JB                 86    0.398  56.490  -0.132   0.622   0.015   0.363
E Y  3Q3JB                 87    0.426  91.015   0.770   0.622   0.015   0.363
B Y  3Q3JB                 88    0.093  19.874   0.122   0.622   0.015   0.363
E D  3Q3JB                 89    0.426  61.459   0.878   0.622   0.015   0.363
E N  3Q3JB                 90    0.344  50.362  -0.079   0.622   0.015   0.363
B V  3Q3JB                 91    0.105  16.077   0.371   0.622   0.015   0.363
B R  3Q3JB                 92    0.178  40.854   0.777   0.430   0.016   0.555
E P  3Q3JB                 93    0.479  67.956  -0.059   0.430   0.016   0.555
B L  3Q3JB                 94    0.217  39.659  -0.370   0.354   0.048   0.598
B C  3Q3JB                 95    0.159  22.267  -0.668   0.278   0.093   0.628
B Y  3Q3JB                 96    0.096  20.472   0.647   0.118   0.150   0.732
E S  3Q3JB                 97    0.411  48.111   0.024   0.053   0.043   0.903
E D  3Q3JB                 98    0.459  66.185  -0.900   0.018   0.019   0.964
B S  3Q3JB                 99    0.031   3.668  -0.053   0.018   0.088   0.893
B D  3Q3JB                100    0.181  26.111   0.229   0.005   0.336   0.660
B A  3Q3JB                101    0.025   2.733   0.689   0.001   0.900   0.099
B V  3Q3JB                102    0.018   2.736   1.316   0.001   0.959   0.040
B L  3Q3JB                103    0.018   3.223   1.644   0.000   0.983   0.017
B L  3Q3JB                104    0.019   3.387   1.690   0.000   0.983   0.017
B C  3Q3JB                105    0.023   3.159   1.184   0.001   0.959   0.040
B F  3Q3JB                106    0.063  12.604   0.967   0.001   0.959   0.040
B D  3Q3JB                107    0.083  12.018  -0.878   0.002   0.816   0.182
B I  3Q3JB                108    0.068  12.506   0.216   0.005   0.336   0.660
E S  3Q3JB                109    0.329  38.582   0.291   0.005   0.045   0.951
E R  3Q3JB                110    0.353  80.837   1.206   0.016   0.005   0.979
E P  3Q3JB                111    0.464  65.870   1.533   0.694   0.003   0.303
E E  3Q3JB                112    0.639 111.598   1.472   0.502   0.002   0.495
B T  3Q3JB                113    0.066   9.196  -0.059   0.694   0.003   0.303
B V  3Q3JB                114    0.061   9.406   0.175   0.923   0.002   0.076
E D  3Q3JB                115    0.579  83.376   1.669   0.923   0.002   0.076
E S  3Q3JB                116    0.388  45.485   1.351   0.923   0.002   0.076
B A  3Q3JB                117    0.018   2.006   1.272   0.970   0.001   0.030
E L  3Q3JB                118    0.349  63.847   1.121   0.970   0.001   0.030
E K  3Q3JB                119    0.593 122.021   1.871   0.970   0.001   0.030
B K  3Q3JB                120    0.139  28.572   1.201   0.970   0.001   0.030
B W  3Q3JB                121    0.058  13.973   0.792   0.970   0.001   0.030
E R  3Q3JB                122    0.418  95.722   1.983   0.970   0.001   0.030
E T  3Q3JB                123    0.448  62.193   1.554   0.988   0.000   0.012
B E  3Q3JB                124    0.046   8.106   0.092   0.970   0.001   0.030
B I  3Q3JB                125    0.203  37.629   0.790   0.970   0.001   0.030
E L  3Q3JB                126    0.654 119.729   1.336   0.923   0.002   0.076
E D  3Q3JB                127    0.534  76.935   0.668   0.782   0.003   0.216
B Y  3Q3JB                128    0.216  46.138  -0.153   0.246   0.004   0.750
E C  3Q3JB                129    0.328  46.107  -0.647   0.016   0.005   0.979
E P  3Q3JB                130    0.719 101.984  -0.002   0.003   0.003   0.994
E S  3Q3JB                131    0.583  68.351   0.466   0.005   0.015   0.979
B T  3Q3JB                132    0.035   4.827   1.293   0.004   0.138   0.858
B R  3Q3JB                133    0.099  22.717   0.864   0.004   0.616   0.381
B V  3Q3JB                134    0.040   6.102   0.738   0.001   0.959   0.040
B L  3Q3JB                135    0.016   2.875   1.704   0.000   0.983   0.017
B L  3Q3JB                136    0.014   2.490   1.746   0.001   0.959   0.040
B I  3Q3JB                137    0.016   2.904   1.371   0.001   0.900   0.099
B G  3Q3JB                138    0.020   1.566   0.214   0.004   0.616   0.381
B C  3Q3JB                139    0.053   7.469   0.392   0.022   0.359   0.619
B K  3Q3JB                140    0.106  21.701   0.433   0.020   0.205   0.775
B T  3Q3JB                141    0.099  13.717   0.095   0.052   0.084   0.864
B D  3Q3JB                142    0.103  14.785  -0.729   0.052   0.084   0.864
B L  3Q3JB                143    0.072  13.275  -0.271   0.113   0.043   0.844
E R  3Q3JB                144    0.400  91.554   0.353   0.257   0.016   0.727
E T  3Q3JB                145    0.605  83.927  -0.410   0.176   0.004   0.820
E D  3Q3JB                146    0.417  60.147  -0.648   0.321   0.003   0.675
E L  3Q3JB                147    0.548 100.357  -1.165   0.522   0.016   0.462
E S  3Q3JB                148    0.599  70.156  -0.632   0.522   0.016   0.462
E T  3Q3JB                149    0.345  47.907  -0.215   0.522   0.016   0.462
E L  3Q3JB                150    0.359  65.696  -0.510   0.622   0.015   0.363
E M  3Q3JB                151    0.519 103.832  -0.179   0.622   0.015   0.363
E E  3Q3JB                152    0.487  85.149  -0.067   0.622   0.015   0.363
E L  3Q3JB                153    0.277  50.664  -0.944   0.522   0.016   0.462
E S  3Q3JB                154    0.524  61.366  -0.896   0.522   0.016   0.462
E H  3Q3JB                155    0.582 105.848  -0.347   0.430   0.016   0.555
E Q  3Q3JB                156    0.605 108.035  -0.739   0.246   0.004   0.750
E K  3Q3JB                157    0.598 122.988  -0.583   0.058   0.017   0.925
E Q  3Q3JB                158    0.600 107.214   0.013   0.018   0.047   0.935
E A  3Q3JB                159    0.396  43.650   0.846   0.019   0.141   0.840
E P  3Q3JB                160    0.502  71.276   1.072   0.004   0.197   0.799
B I  3Q3JB                161    0.040   7.326   0.311   0.004   0.138   0.858
E S  3Q3JB                162    0.390  45.720   1.539   0.018   0.047   0.935
E Y  3Q3JB                163    0.409  87.339   1.051   0.923   0.002   0.076
E E  3Q3JB                164    0.722 126.168   1.240   0.970   0.001   0.030
E Q  3Q3JB                165    0.352  62.867   1.513   0.970   0.001   0.030
B G  3Q3JB                166    0.027   2.141  -0.227   0.970   0.001   0.030
E C  3Q3JB                167    0.373  52.327   2.024   0.970   0.001   0.030
E A  3Q3JB                168    0.565  62.296   1.957   0.970   0.001   0.030
B I  3Q3JB                169    0.098  18.149   1.299   0.970   0.001   0.030
B A  3Q3JB                170    0.019   2.105   0.156   0.970   0.001   0.030
E K  3Q3JB                171    0.679 139.609   1.641   0.970   0.001   0.030
E Q  3Q3JB                172    0.650 116.054   1.388   0.923   0.002   0.076
B L  3Q3JB                173    0.231  42.259   1.282   0.600   0.003   0.397
E G  3Q3JB                174    0.719  56.609   0.226   0.016   0.005   0.979
B A  3Q3JB                175    0.083   9.191   0.027   0.005   0.015   0.979
E E  3Q3JB                176    0.277  48.427   0.377   0.020   0.205   0.775
B I  3Q3JB                177    0.174  32.190   1.087   0.021   0.756   0.223
B Y  3Q3JB                178    0.050  10.600   0.577   0.018   0.846   0.136
B L  3Q3JB                179    0.057  10.437   0.334   0.018   0.846   0.136
B E  3Q3JB                180    0.054   9.364  -0.324   0.018   0.846   0.136
B G  3Q3JB                181    0.018   1.440   1.187   0.023   0.655   0.322
B S  3Q3JB                182    0.046   5.344   0.248   0.069   0.386   0.545
B A  3Q3JB                183    0.035   3.879  -1.685   0.216   0.235   0.548
E F  3Q3JB                184    0.249  49.894   0.499   0.184   0.043   0.773
E T  3Q3JB                185    0.352  48.864  -0.302   0.115   0.016   0.868
E S  3Q3JB                186    0.451  52.810  -1.316   0.109   0.005   0.886
E E  3Q3JB                187    0.535  93.465   0.068   0.406   0.004   0.590
E K  3Q3JB                188    0.376  77.302   1.478   0.502   0.002   0.495
B S  3Q3JB                189    0.161  18.822   0.851   0.181   0.016   0.803
B I  3Q3JB                190    0.025   4.551   1.191   0.923   0.002   0.076
E H  3Q3JB                191    0.423  76.889   2.199   0.970   0.001   0.030
E S  3Q3JB                192    0.396  46.470   1.965   0.970   0.001   0.030
B I  3Q3JB                193    0.026   4.791   1.382   0.988   0.000   0.012
B F  3Q3JB                194    0.021   4.235   1.349   0.988   0.000   0.012
E R  3Q3JB                195    0.317  72.570   2.067   0.988   0.000   0.012
E T  3Q3JB                196    0.299  41.513   2.118   0.988   0.000   0.012
B A  3Q3JB                197    0.025   2.744   1.142   0.988   0.000   0.012
B S  3Q3JB                198    0.022   2.602   0.900   0.988   0.000   0.012
E M  3Q3JB                199    0.399  79.860   2.194   0.988   0.000   0.012
E L  3Q3JB                200    0.267  48.924   2.068   0.988   0.000   0.012
B C  3Q3JB                201    0.026   3.580   0.636   0.970   0.001   0.030
B L  3Q3JB                202    0.163  29.864   0.845   0.970   0.001   0.030
E N  3Q3JB                203    0.650  95.204   1.737   0.923   0.002   0.076
E K  3Q3JB                204    0.432  88.821   1.487   0.782   0.003   0.216
E P  3Q3JB                205    0.411  58.363  -0.042   0.694   0.003   0.303
E S  3Q3JB                206    0.679  79.637   0.259   0.502   0.002   0.495
E P  3Q3JB                207    0.655  92.944   0.660   0.406   0.004   0.590
E L  3Q3JB                208    0.549 100.449   0.241   0.246   0.004   0.750
E P  3Q3JB                209    0.649  92.150   0.339   0.176   0.004   0.820
E Q  3Q3JB                210    0.724 129.324   1.144   0.109   0.005   0.886
E K  3Q3JB                211    0.689 141.768   0.625   0.053   0.005   0.942
E S  3Q3JB                212    0.633  74.199   0.082   0.016   0.005   0.979
E P  3Q3JB                213    0.543  77.123  -1.198   0.016   0.005   0.979
E V  3Q3JB                214    0.693 106.560  -1.413   0.000   0.001   0.999

Explain the output.  Go back.
```

---
